# Causes of Chronic Kidney Disease in Iranian Children: A Meta-Analysis and Systematic Review

**DOI:** 10.5334/aogh.2391

**Published:** 2019-03-13

**Authors:** Hosien Shahdadi, Mahmood Sheyback, Hosein Rafiemanesh, Abbas Balouchi, Salehoddin Bouya, Gholamhossein Mahmoudirad

**Affiliations:** 1 Department of Nursing, Faculty of Nursing and Midwifery, Zabol University of Medical Science, Zabol, IR; 2 Student Research Committee, School of Nursing, Bushehr University of Medical Sciences, Bushehr, IR; 3 Student Research Committee, Department of Epidemiology, School of Public Health and Safety, Shahid Beheshti University of Medical Sciences, Tehran, IR; 4 Nursing, Student Research Committee, Nursing and Midwifery School, Iran University of Medical Sciences, Tehran, IR; 5 Internal Medicine and Nephrology, Clinical Immunology Research Center, Ali-Ebne Abitaleb Hospital, Zahedan University of Medical Sciences, Zahedan, IR; 6 Nursing Department, Faculty of Nursing and Midwifery, Birjand University of Medical Sciences, Birjand, IR

## Abstract

This study aimed to determine the causes of chronic kidney disease (CKD) in Iranian children. In this systematic review and meta-analysis study, international (PubMed, Web of Science, Scopus, and Google Scholar) and national (SID, Magiran) databases were searched for articles published through December 30, 2017. The quality of the studies was determined using the Hoy instrument. Out of 2,117 initial studies, 13 studies performed on a total of 3,596 children were included in the final stage of the study. Based on the results of the random effects method (95% CI), the main causes of chronic kidney disease in stages 1–4 (CKD) were CAKUT (37%) and glomerulonephritis (19.96%); in stage 5 (ESRD) they were CAKUT (40.82%) and urological disorders (27.44%). Considering the high prevalence of CAKUT, glomerulonephritis and urinary problems, the use of comprehensive approaches can be very effective in enhancing the knowledge of patients about the causes of kidney disease. The results obtained from the present study can assist policymakers in more accurately planning screenings of the causes of CKD in Iranian children.

## 1. Introduction

Chronic kidney disease (CKD) is one of the fastest growing chronic diseases, and today it is a major health problem worldwide [1]. According to the Global Burden of Disease Study (2010), CKD shifted from the 36th leading cause of death in 1990 to the 19th leading cause in 2013 [2]. The care associated with advanced stages of CKD impose staggering costs upon healthcare systems, even in advanced countries. For example, in the United States, 6.3% of the annual Medicare budget is spent on care for CKD patients [3]. Over 500 million people around the world suffer from CKD, with more than 80% of them living in developing countries [4]. There is no precise information about the global prevalence of CKD in children; however, studies show that in Europe, there are 11 to 12 cases per million of age-related population (pmarp) [5,6]. CKD in children is associated with cardiovascular complications, and the identification of the causes, especially in children, help improve treatment and prevent progression of the disease [7]. The causes of CKD in children are very different from those in adults [5,8].

According to North American Pediatric Renal Trials and Collaborative Studies (NAPRTCS) and Europe, the most common causes of CKD are congenital anomalies of the kidney and urinary tract (CAKUT) (48–58%) and hereditary nephropathy (10–19%) [6,9,10]. Due to the absence of valid registries in Asian countries, obtaining a precise determination of the causes is difficult, but individual studies have shown that the most common cause in countries including Turkey and Kuwait include CAKUT (47–62%) and obstructive uropathy following hereditary nephropathy (17–30%) [11,12]. However, in India, glomerulonephritis is the most common cause of CKD [13].

The causes of CKD have differed across individual studies conducted in Iran [14,15,16]. There is no comprehensive evidence for the exact share of each cause in Iran. Unfortunately, unlike adults, very few epidemiological studies have been performed on a population of children [5,17]. As the causes of disease can affect treatment procedures, their early identification in CKD in children helps in the better management of the disease, early detection of preventable and non-preventable causes, better prediction of prognosis and consultation with afflicted children and their families, prevention of progression of the disease causes, and a reduction in cardiovascular complications resulting in diminished patient mortality. Furthermore, identifying the causes leads to better management of symptoms by families and will assist health policymakers reduce the costs incurred to the health system of Iran (as a developing country), where a large portion of the health system resources is allocated to chronic diseases, such as CKD. This study aimed to identify the causes of CKD in Iranian children as an understudied population.

## 2. Methods

### 2.1. Eligibility Criteria

The protocol has been published (PROSPERO: CRD42018087316), and the methods adopted for this systematic review were developed in accordance with the Cochrane Handbook for Systematic Reviews and reported using the Preferred Reporting Items for Systematic Reviews and Meta-Analyses (PRISMA) tool [18]. Observational studies conducted on children populations in different CKD stages (1–5) living in Iran were included. Outcomes as reported in the studies were collected. The minimum required sample size was 25 patients in every study. The causes of CKD measured in the current study were defined according to the KDOQI criteria as different categories, such as glomerulonephritis, congenital anomalies of the kidney and urinary tract (CAKUT), urological disorders, urinary calculi, and unknown [19].

### 2.2. Search strategy

International (PubMed, Google scholar, Web of science and Scopus) and national (SID and Magiran) databases and a key national journal (Iranian Journal of Kidney Diseases) were searched for relevant studies without settings and language limits from launching to 30 December 2017. The MEDLINE search strategy was adopted to search other databases. The specific search strategies were created by a health sciences librarian with expertise in systematic review according to the PRESS standard [20]. Boolean operators (AND, OR, and NOT), Medical Subject Headings (MeSH), truncation “*” and related text words were used for searching in titles and abstracts. Keywords used were “Causes” OR “kidney disease” OR “kidney failure” OR “kidney insufficiency” OR “renal disease” OR “renal failure” OR “renal insufficiency” OR “chronic kidney disease” OR “CKD” OR “children” OR “pediatrics” OR “Iran.”

### 2.3. Selection of studies and data extraction

In accordance with the study protocol, two researchers independently screened titles and abstracts related to the eligibility criteria. In the next step, duplicated studies were removed, full texts were screened for eligibility criteria, and the required information was extracted. The consensus method was used to solve controversies between the two researchers. Extracted data items included first author (year), province, design, stage of CKD, setting, sample size, sampling method, study duration, risk of bias, patient age, patient gender, and causes of CKD in children.

### 2.4. Quality assessment

To assess the methodological quality and risk of bias of each included observational study, all studies were evaluated using the tool introduced by Hoy et al. This 10-item tool evaluated the quality of studies in the two dimensions of external validity (items 1–4 assessed target population, sampling frame, sampling method, and minimal nonresponse bias) and internal validity (items 5–9 assessed data collection method, case definition, study instrument, and mode of data collection; item 10 assessed bias related to analysis). Risk of bias was evaluated by two researchers independently; Disagreements were resolved through the consensus method.

### 2.5. Data synthesis

All eligible studies were included in the synthesis after a systematic review. Data was combined with the forest plot. The causes of CKD in children were evaluated by the random-effects model. The heterogeneity of the preliminary studies was evaluated with I2 tests and univariate meta regression model. Sub-group analysis was conducted to determine heterogeneity based on the study gender, age and community location (rural/urban). Meta-analysis was performed using STATA 14 (StataCorp, Texas, USA) statistical software.

## 3. Results

### 3.1. Overall results

#### 3.1.1. Study selection

**Figure 1:**
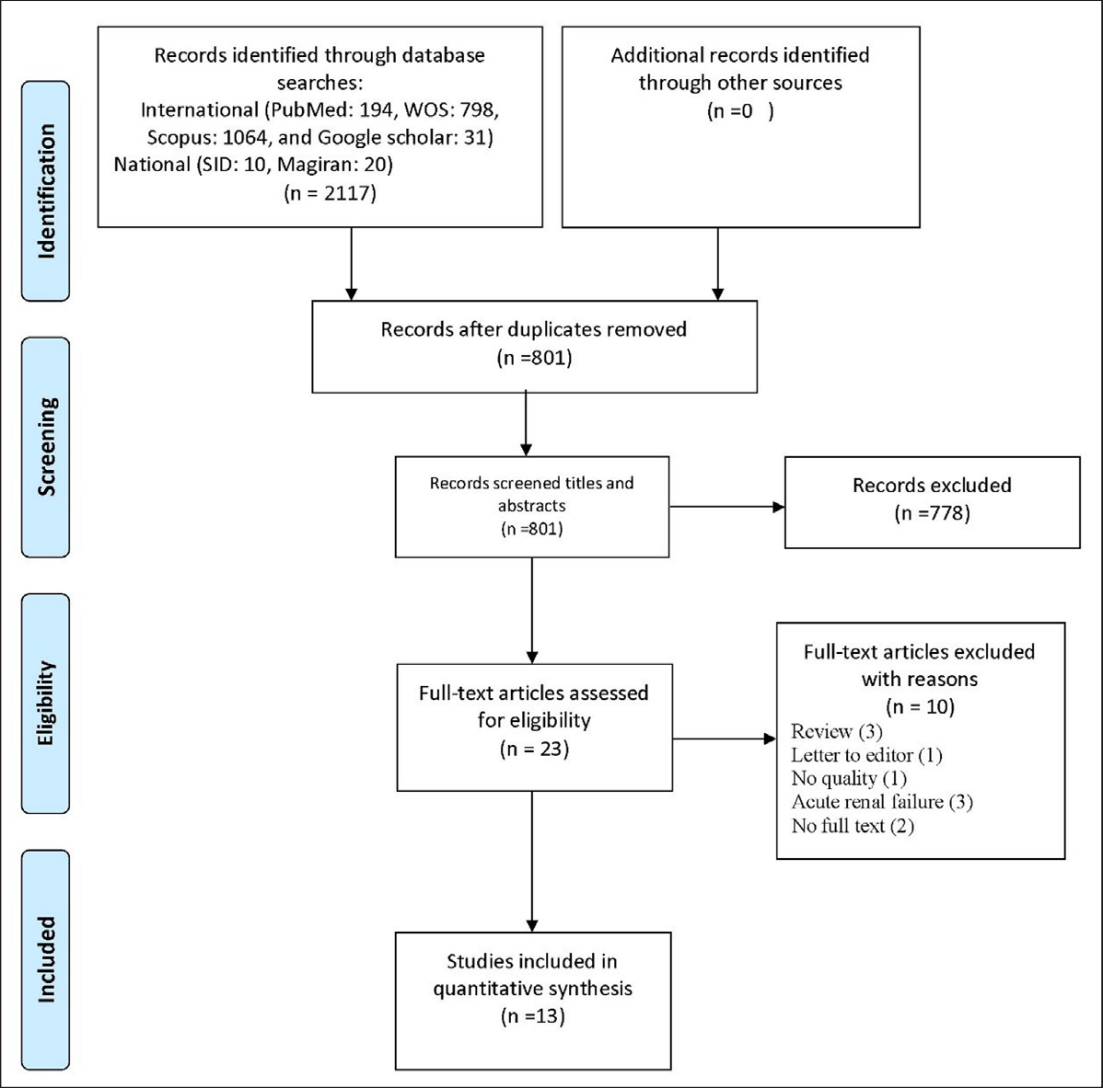
Flowchart of study selection process.

A total of 2,117 articles from the initial searches were retrieved from various databases. Out of 801 non-duplicate studies in the title and abstract screening process, 778 studies were omitted due to lack of relevance. Of the remaining 23 studies, 13 met the eligibility criteria. Of 10 excluded studies, three were reviews, one was a letter to the editor, three were conducted on acute renal failure patients, one had no full text available, and two did not meet the minimum quality requirements for inclusion in the study (Figure 1).

#### 3.1.2. Study characteristics

The studies included in this review were conducted on a total of 3,596 Iranian children. The ages of participants ranged from 1 to 17 years. Of the 13 included studies, 5 were cross-sectional and 7 were retrospective studies. There were 13 studies obtained from 7 provinces: 4 from Tehran [14,21,22,23], 2 from Fars [16,24], 2 from Isfahan [25,26], and 2 from East Azerbaijan [27,28]. One study was conducted in each of the provinces of Khuzestan [29], Khorasan Razavi [15], and Mazandaran [30]. The sampling method used in all studies was census. Most studies (9) had a low risk of bias [14,15,21,22,23,25,26,28,29,30]. The setting in most of the studies (12) was a hospital. Study duration ranged from 1 to 24 years. CKD stages in most studies (11) were stages 1–4 (Table 1).

**Table 1:** Summary of included studies.

First Author (year)	Province	Design	Stage of CKD	Setting	Sample Size	Study Duration	Risk of bias

Ahmadzadeh, A., et al. (2009) [29]	Khuzestan	Retrospective	Stage 1–4	Hospital	139	10(y)	Low
Ataei, N., et al. (2016) [14]	Tehran	Retrospective	Stage 1–5	Hospital	363	24(y)	Moderate
Azarfar, A., et al. (2017) [15]	Khorasan Razavi	Cross-sectional	Stage 1–5	Hospital	326	7(y)	Low
Derakhshan, A., et al. (2003) [16]	Fars	Retrospective	Stage 1–4	Hospital	1358	6.5(y)	Low
Fallahzadeh, M. H. (2001) [24]	Fars	Prospective	Stage 1–4	Hospital	170	10(y)	Moderate
Gheissari, A., et al. (2012) [25]	Isfahan	Retrospective	Stage 1–4	Hospital	268	11(y)	Low
Gheissari, A., et al. (2013) [26]	Isfahan	Cross-sectional	Stage 1–5	School	712	1(y)	Low
Madani, K., et al. (2001) [23]	Tehran	Cross-sectional	Stage 1–4	Hospital	166	9(y)	Low
Mortazavi, F., et al. (2006) [27]	East Azerbaijan	Retrospective	Stage 1–4	Hospital	55	4.5(y)	Moderate
Mortazavi, F., et al. (2010) [28]	East Azerbaijan	Retrospective	Stage 1–5	Hospital	115	10(y)	Low
Otukesh, H., et al. (2001) [21]	Tehran	Retrospective	Stage 1–4	Hospital	245	1(y)	Moderate
Sharifian, M., et al. (2016) [22]	Tehran	Cross-sectional	Stage 1–5	Hospital	104	1(y)	Low
Sorkhi, H. (2001) [30]	Mazandaran	Cross-sectional	Stage 1–5	Hospital	85	1(y)	Low

#### 3.1.3. Causes of chronic kidney diseases in children

This meta-analysis was done on 13 studies conducted on 3,596 Iranian children. Based on the results of the random effect method, the main causes of CKD were CAKUT, urological disorders, and glomerulonephritis, with pooled effect sizes of 38.40% (I^2^ = 99.33%), 22.28% (I^2^ = 97.84%), and 18.21% (I^2^ = 96.39%), respectively. In the sub-group analysis based on stages of CKD, the results showed in stages 1–4, CAKUT was the more prevalent cause of CKD and in stage 5 (ESRD), it was glomerulonephritis. The prevalence of an unknown cause in Stage 1–4 of CKD was more than twice that of the stage 5 (ESRD) (Table 2).

**Table 2:** Prevalence of causes based on stage of CKD.

First author (Year)	Glomerulonephritis		CAKUT		Urological disorders		Urinary calculi		Unknown	

	ES (95% CI for ES)	Weight	ES (95% CI for ES)	Weight	ES (95% CI for ES)	Weight	ES (95% CI for ES)	Weight	ES(95% CI for ES)	Weight

**Stage 1–4**										

**Ahmadzadeh, A.(2009)** [29]	6.47 (3.44, 11.85)	7.94	67.63 (59.46, 74.84)	9.06					10.79 (6.65, 17.04)	10.03
**Derakhshan, A. (2003)** [16]	22.97 (20.82, 25.29)	8.14	0.96 (0.56, 1.63)	9.23	7.58 (6.29, 9.12)	25.91				
**Fallahzadeh, M.H. (2001)** [24]	15.29 (10.66, 21.47)	7.74	15.29 (10.66, 21.47)	9.15			24.12 (18.30, 31.07)	29.63	13.53 (9.19, 19.48)	10.06
**Gheissari, A. (2012)** [25]	42.57 (35.96, 49.47)	7.48	34.16 (27.97, 40.94)	9.11						
**Madani, K. (2001)** [23]	10.24 (6.49, 15.79)	7.87	46.99 (39.55, 54.56)	9.07					8.43 (5.09, 13.66)	10.45
**Mortazavi, F. (2006)** [27]	23.48 (16.67, 32.0)	7.29	58.26 (49.12, 66.86)	9.01			2.61 (0.89, 7.39)	34.69	9.57 (5.43, 16.32)	9.92
**Otukesh, H. (2001)** [21]	20.41 (15.84, 25.89)	7.80	36.73 (30.95, 42.93)	9.13					3.67 (1.94, 6.83)	11.11
**Sub-total random pooled ES**	**19.96 (12.61, 27.31)**	**54.26**	**37.00 (15.63, 58.36)**	**63.76**					**8.84 (4.91, 12.77)**	**51.53**
**Stage 5 (ESRD)**										

**Ataei, N. (2016)** [14]	20.11 (16.31, 24.54)	7.94	59.50 (54.38, 64.43)	9.16					19.56 (15.81, 23.95)	10.51
**Azarfar, A. (2017)** [15]	3.68 (2.12, 6.32)	8.16			38.34 (33.23, 43.73)	25.30	2.76 (1.46, 5.16)	35.68	20.86 (16.80, 25.60)	10.37
**Gheissari, A. (2013)** [26]	35.07 (29.61, 40.96)	7.69	34.33 (28.90, 40.20)	9.14					21.64 (17.13, 26.95)	10.13
**Mortazavi, F. (2010)** [28]	25.45 (15.81, 38.3)	6.40	61.82 (48.61, 73.48)	8.78					12.73 (6.30, 24.02)	8.12
**Sharifian, M. (2016)** [22]	8.65 (4.62, 15.63)	7.74	8.65 (4.62, 15.63)	9.15	14.42 (8.94, 22.44)	24.91				
**Sorkhi, H. (2001)** [30]	5.88 (2.54, 13.04)	7.81			29.41 (20.79, 39.82)	23.89			10.59 (5.67, 18.91)	9.33
**Sub-total random pooled ES**	**16.13 (6.28, 25.98)**	**45.74**	**40.82 (15.40, 66.24)**	**36.23**	**27.44 (11.78, 43.11)**	**74.09**			**17.94 (14.10, 21.78)**	**48.47**
**Random pooled ES**	**18.21 (12.04, 24.39)**	**100**	**38.40 (21.19, 55.61)**	**100**	**22.28 (5.51, 39.06)**	**100**	**9.04 (0.87, 17.20)**	**100**	**13.11 (8.44, 17.78)**	**100**

**Figure 2:**
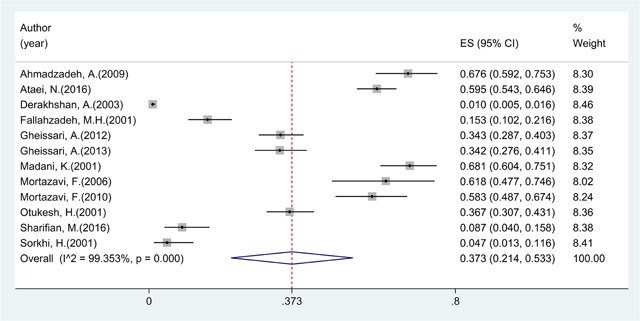
Prevalence of CAKUT in Iranian children.

**Figure 3:**
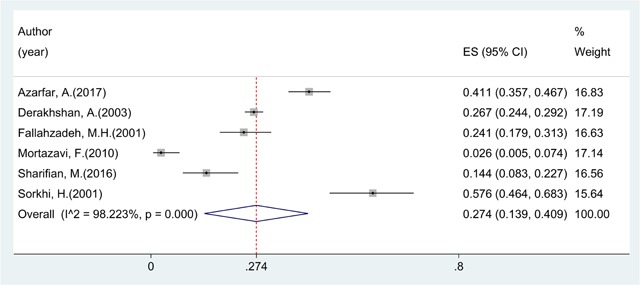
Prevalence of urological disorders in Iranian children.

Overall prevalence of CAKUT was 37.32% (95% CI: 21.38, 53.25; I^2^ = 99.35%) (Figure 2) and of urological disorders was 27.38% (95% CI: 13.90, 40.86; I^2^ = 98.22%) (Figures 2 and 3).

#### 3.1.4. Meta-regression

**Figure 4:**
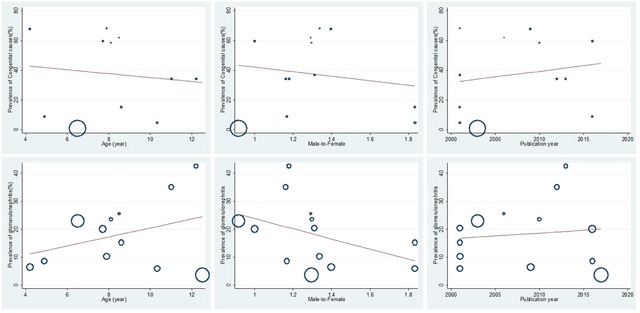
Meta-regression of age, gender (male-to-female ratio), year of publication, and prevalence of congenital causes (CAKUT) and glomerulonephritis in Iranian children.

The results of univariate meta-regression analyses showed that participant’s mean age, gender (male-to-female ratio), and year of publication did not contribute significantly to the heterogeneity of CAKUT and glomerulonephritis (p-value > 0.05) (Figure 4).

## 4. Discussion

### 4.1. Causes of CKD among children

This systematic review of publications through 30 December 2017 aimed to determine the causes of CKD in Iranian children. A total of 13 studies conducted on a total of 3,596 Iranian children were included in the meta-analysis. The overall main causes were CAKUT (38.4%), urological disorders (22.28%), and glomerulonephritis (14%). The results of sub-group analysis of stages 1–4 (CKD) and stage 5 (ESRD) showed that the main causes in stages 1–4 of CKD were CAKUT (37%) and glomerulonephritis (19.96%).

Due to a shortage of information on the CKD epidemiology in children, no study was found that examined the causes of CKD in children around the world through a meta-analysis. Registry-based studies have indicated that the most common causes of CKD in the U.S. were CAKUT (48%), miscellaneous (21%), and glomerulonephritis (14%) (US) [9]; in Italy, they were CAKUT (58%), hereditary nephropathy (15%), and miscellaneous (10%) [6]; and in Belgium, they were CAKUT (59%), hereditary nephropathy (19%), and cystic kidney disease (9%) [10]. As with the present study, all other studies identified CAKUT as the most important cause of CKD in children around the world. Other important causes in Iran were included: glomerulonephritis and hereditary nephropathy, which indeed have been placed in the classification of CAKUT in the present study and are considered important causes of CKD in children.

Causes of CKD in different regions of Iran were different, which may be due to different levels of health literacy among the people in the studies. The most common cause of CKD was CAKUT, which showed genetic diseases, including hereditary diseases and congenital malformations, are an important cause of the high incidence of CKD among Iranian children. Another important cause of the high incidence of CAKUT is the lateness of visits and referrals to the nephrologist due to latent CKD progress and high rates of consanguineous marriage [26,31].

Unlike the present study, CKD in children in Italy and Belgium had miscellaneous causes [6,10]. Furthermore, the prevalence of each cause differed from country to country, suggesting a difference in the pattern of causes of CKD across different countries. In the East Mediterranean Region, such as Turkey, CAKUT is again the most important cause of CKD in children, confirming the results of the present study, but due to some differences between Iran and Turkey, we should interpret them with caution [12]. Although the general pattern is the same across various countries, the prevalence of each cause is different. In this regard, the prevalence of CAKUT in American and European countries [6,10] was 48–59%, but in the present study, the prevalence of congenital causes and CAKUT was around 37%. The prevalence of causes leading to nephropathy, including glomerulonephritis and hereditary causes, in European countries and the U.S. was 10–14% [6,9,10]. However, in the present study, the prevalence of glomerulonephritis was 19.96%, which may be due to the methodology of the studies, including the place where the research was conducted, sample size, sampling method, or the socioeconomic causes of health status, including a poor level of knowledge about CKD among the parents of children, less access to health services, and delayed referral to a nephrologist. The high prevalence of glomerulonephritis could suggest a greater prevalence of viral and bacterial infections in developing countries, such as Iran [32,33,34].

The results also revealed that in the ESRD group (stage 5), the most common causes of CKD were CAKUT (40.82%) and urological disorders (27.44%). Studies have indicated that the causes of CKD prevalence in children in the ESRD stage in Australia and New Zealand include CAKUT (34%) and glomerulonephritis (29%) [35], CAKUT (36%) and hereditary nephropathy (22%) in Europe [36], CAKUT (43%) and glomerulonephritis (18%) in England [37], and CAKUT (36%) and glomerulonephritis (22%) in Asian countries, including Japan [38]. As with the present study, CAKUT was identified by other studies as the most common cause of CKD in children at the ESRD stage. The prevalence of CAKUT is higher in Iran than in other Asian counties, such as Japan, which may suggest a higher rate of genetic diseases, late diagnosis of pediatric diseases, and poorer health conditions in Iranian children compared to Japanese children.

### 4.2. Limitations

The limitations of the present study were different classifications of CKD causes in some included studies, a lack of information in some studies where the authors were contacted, and the design type of studies, most of which were retrospective. Another limitation was the scope of the studies, which were performed in only 7 provinces (from a total of 31 provinces). One limitation was the target population; we included studies conducted on Iran as the study aim, but it was better for determining the CKD causes in the Middle East and children around the globe. The most important limitation of the present study was a majority of the studies were conducted in hospital settings, which may affect the generalizability of results. It seems due to the lack of a national screening program for early detection of CKD, a lack of community-based CKD related research and necessary required infrastructure, and the low number of community-based research centers in Iran, despite a lot of progress in national medical research toward general population health concerns in the last decade, the main focus of research is on hospitalized patients. The planning and implementation of programs related to early detection of CKD in children can lead to prevention and the minimization of costs for managing ESRD in child patients.

### 4.3. Strengths

To the best of the authors’ knowledge, the current study is the first systematic review and meta-analysis on this subject conducted in Iran. Moreover, considering the shortage of epidemiological studies related to CKD causes around the world, this study can be used as sample evidence in the region. The report of the causes given the stage of CKD was another strength of this study.

## 5. Conclusion

This systematic review and meta-analysis showed that, as with the global pattern, the most important causes of CKD in Iranian children include CAKUT, urological disorders, and glomerulonephritis; the prevalence of these causes, however, is greater among Iranian children, suggesting the importance of paying attention to early screenings in the pre-natal period and early and timely screenings at birth for early detection and management of CKD during childhood. In addition, considering the high prevalence of glomerulonephritis and urinary problems in Iran, the use of a multidimensional approach can be effective in enhancing the knowledge of patients about the causes of kidney diseases. The results obtained from the present study can assist policymakers in more accurately planning screenings of CKD causes in Iranian children. Due to the majority of included studies that were hospital based, we should use the results with caution. Regarding the limited number of provinces involved in the studies, conducting further research emphasizing the inclusion of all provinces can contribute to a better understanding and more accurate detection of the causes of CKD in children.
